# Diagnostic accuracy of endoscopic findings for cytomegalovirus reactivation in hospitalized patients with ulcerative colitis

**DOI:** 10.1371/journal.pone.0331695

**Published:** 2026-02-06

**Authors:** Mamiko Aoi, Naohiro Nakamura, Yusuke Honzawa, Norimasa Fukata, Makoto Naganuma

**Affiliations:** Third Department of Internal Medicine, Division of Gastroenterology and Hepatology, Kansai Medical University, Hirakata, Osaka, Japan; Debre Markos University, ETHIOPIA

## Abstract

**Background:**

Although cytomegalovirus (CMV) reactivation is implicated in ulcerative colitis (UC) exacerbation, the efficacy of antiviral treatment in hospitalized patients with UC with suspected CMV infection has not been thoroughly investigated. We aimed to investigate the diagnostic accuracies of typical endoscopic findings for CMV reactivation in hospitalized UC patients.

**Methods:**

A total of 143 hospitalized cases due to the exacerbation of UC were retrospectively collected. Sensitivity, specificity and diagnostic accuracy of endoscopic findings (punched-out ulcer, round ulcer, girdle ulcer, longitudinal ulcer, and ulcer with wide mucosal defect)for prediction of CMV colitis (histological CMV positivity) and CMV viremia (antigenemia, serum DNA) was assessed. Endoscopic characteristics were compared between patients who initially received anti-viral treatment and who did not receive.

**Results:**

The diagnostic performance of endoscopic findings for histologically confirmed CMV infection varied by lesion type. Punched-out ulcers demonstrated a sensitivity of 66.7%, specificity of 58.3%, and overall diagnostic accuracy of 59.1%. Longitudinal ulcers showed a sensitivity of 58.3%, specificity of 40.0%, and diagnostic accuracy of 41.7%. In contrast, wide mucosal defects exhibited lower sensitivity (16.6%) but higher specificity (75.0%), with an overall diagnostic accuracy of 69.7%. The same tendencies were found as diagnostic accuracies of endoscopic findings of punched-out ulcers, longitudinal ulcers, and wide mucosal defects for CMV antigenemia positivity was 55.6%, 40.0%, 60.4%, respectively. The specificity of girdle ulcers for positivity of serum DNA test was relatively high (84.8%) while sensitivity was 9.1%. In total, the diagnostic accuracies of endoscopic findings with punched-out, round, girdle, and longitudinal ulcers, and those with wide mucosal defects were 46.9%, 42.9%, 33.7%, 51.0%, and 35.7% for positivity of serum DNA test, respectively. Punched-out ulceration (57% vs.13%; p < 0.001), longitudinal ulceration (70%vs.34%; p < 0.001), and ulceration with wide mucosal defects (31%vs.9%; p < 0.001) were higher with ganciclovir treatment than without.

**Conclusion:**

Endoscopic findings cannot predict CMV antigenemia or CMV colitis. Therefore, antiviral treatment should not be administered without evidence of CMV reactivation using only endoscopic findings.

## Introduction

Ulcerative colitis (UC) is characterized by periods of remission and relapse; however, the etiology and associated morbidity remain unknown. However, its pathophysiology has been extensively studied, and medical treatments have been developed recently [1]. Conventionally, most patients with mild to moderate UC are treated with 5-aminosalicylates, whereas corticosteroid (CS) therapy is one of the most effective treatments for moderate to severe UC. Intravenous CS is the first-line treatment for hospitalized patients with severe acute UC [2–5]. Although the short-term efficacy of CS in hospitalized patients is high [6,7], some patients develop resistance to CS [8]. Short-term colectomy rates of 25–30% have been reported in patients with acute severe UC [9].

Among patients with steroid-resistant UC, cytomegalovirus (CMV) reactivation aggravates the disease [10–11]. CMV is a common virus belonging to the herpes virus family, and approximately 70–80% of adults are infected with CMV during childhood. Most individuals do not experience symptoms under normal immune conditions, but reactivation of CMV causes morbidity and non-relapse mortality in states of immune deficiency, such as post-hematopoietic cell transplantation and human immunodeficiency virus infection [12]. In UC, CMV is reactivated when using CS or immunosuppressants, or when colonic inflammation is severe, even in patients without immunosuppressive agents. Recent British guidelines indicate that CMV reactivation in the colonic mucosa of patients with exacerbated UC or Crohn’s disease may be treated with intravenous ganciclovir [3]. They also recommended that colonic biopsy should be performed to diagnose CMV disease, preferably by immunohistochemistry because the CMV antigenemia test and blood PCR exhibit poor sensitivity in diagnosing CMV colitis [13,14].

However, confirmation of CMV colonic infections using immunohistochemistry or quantitative tissue PCR in hospitalized patients with UC may take time. Therefore, previous studies have been conducted to identify clinical and endoscopic features associated with CMV reactivation. Suzuki et al. demonstrated that specific endoscopic features, such as irregular ulceration, wide mucosal defects, punched-out ulceration, and longitudinal ulceration, were associated with CMV reactivation [15]. Hirayama et al. also reported that the presence of punched-out ulcers was associated with CMV infection in patients with active UC [16]. Yang et al. also found that punch-out ulcers and high CMV viremia were associated with the presence of CMV colitis on histological examination [17].

However, the validity of these endoscopic characteristics has not been established for CMV infection. In this study, diagnostic accuracy of endoscopic findings for CMV reactivation on CMV antigenemia, immunohistochemistry or serum colonic DNA positive was assessed in hospitalized patients with UC.

## Methods

### Patients

Data of hospitalized patients who were suspected CMV reactivation for UC exacerbation between 2006 and 2022 were retrospectively collected from the medical charts at Kansai Medical Hospital. These cases were mainly treated with antiviral treatments (ganciclovir). Also, hospitalized patients who received medical treatment for UC without ganciclovir (non-ganciclovir group) were also collected to compare the differences in clinical and endoscopic characteristics between patients treated with ganciclovir and those who did not receive ganciclovir. Ganciclovir was administered at a daily dose of 5 mg/kg body weight for 14 days. All patients with UC were diagnosed according to the guidelines of the Research Committee on Inflammatory Bowel Disease in Japan.

### Data collection

We assessed demographic data at entry, sex, age, disease duration, extent of disease (pancolitis, left side colitis, proctitis), blood biomarkers (hemoglobin, platelet count, serum albumin, C-reactive protein (CRP) level, and erythrocyte sedimentation rate), Patients Report Outcome 2 (PRO2), Mayo endoscopic subscore (MES) [18], the Ulcerative Colitis Endoscopic Index of Severity (UCEIS) [19,20], medical treatment for the UC exacerbation during hospitalization. PRO2 was defined as the sum of all Mayo sub-scores for daily diarrhea and rectal bleeding. All data was collected from medical charts from January 2023 to June 2024. Authors had access to information that could identify individual participants during or after data collection.

### Assessment of endoscopic characteristics

Endoscopic activity and patient characteristics before ganciclovir or medical treatment for UC were assessed. Endoscopic severity was prospectively evaluated according to the Mayo Endoscopic Subscore (MES) and Ulcerative Colitis Endoscopic Index of Severity (UCEIS). The MES was classified as MES0 (endoscopic remission), MES1(mild mucosal inflammation), MES2 (moderate mucosal inflammation), and MES3 (severe mucosal inflammation). The UCEIS was scored using the following items: vascular pattern (three levels), bleeding (four levels), erosions, and ulcers (four levels) and ranged from 0 (normal) to 8 (severe). The most severely affected area of the mucosa was scored [19,20].

All endoscopic findings were evaluated by an expert endoscopist (MN; gastrointestinal [GI] physician with 30 years of experience, who performs >100 endoscopic procedures in inflammatory bowel disease cases annually for 20 years) and a non-expert endoscopist for inflammatory bowel disease (MA; GI physician with 15 years of experience). Both endoscopists discussed and mutually decided on each item.

**Fig 1:**
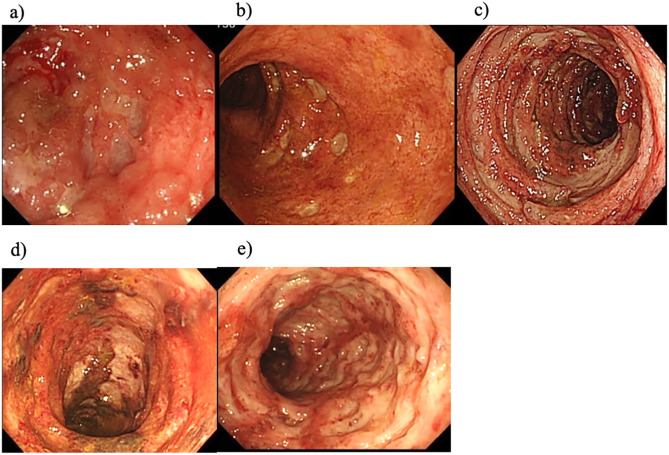
Typical endoscopic findings: a) punched out ulcer, b) round/oval ulcer, c) girdle ulcer, d) longitudinal ulcer, and e) wide mucosal defect.

To assess the endoscopic characteristics, the morphology of ulceration was evaluated to determine the indications for ganciclovir. The presence of punched, longitudinal, round and annular/ girdle ulcerations, as well as ulceration with wide mucosal defects were evaluated. Typical endoscopic findings are shown in Fig 1.

### Assessment for CMV reactivation

CMV reactivation was diagnosed based on the presence of antigenemia for CMV pp65 (C7HRP) (SRL Inc. Tokyo, Japan), typical histological findings (the presence of either colonic mucosal inclusion bodies or positive cells by immunohistochemistry), and positivity for serum CMV immunoglobulin G antibodies. DNA qualitative test for CMV was also assessed in some patients of this cohort (SRL Inc. Tokyo, Japan).

### Endpoints and statistical analysis

Baseline characteristics of patients were described using means with standard deviations for continuous data and absolute numbers with percentages for categorical data. The primary endpoint was correlations between CMV positivity and endoscopic findings (punched-out ulcers, round ulcers girdle ulcers, longitudinal ulcers, wide mucosal defect) was assessed. The diagnostic accuracies (sensitivity, specificity, positive predictive value (PPV), negative predictive value (NPV)) of endoscopic findings for positivity of CMV antigenemia, histological CMV detection, DNA qualitative test) were analyzed.

The main secondary endpoints were comparing endoscopic characteristics between patients who received ganciclovir (ganciclovir group) and those who were not administered ganciclovir during hospitalization (non-ganciclovir group). Categorical variables were compared using the chi-square or Fisher’s exact test. Paired variables were evaluated using the Mann–Whitney U test or Kruskal–Wallis test.

All statistical analyses were performed using the IBM SPSS Statistics version 26 (IBM Corp., Armonk, NY, USA).

### Ethics

The study design was reviewed and approved by the Ethics Committee of the Kansai Medical University Hospital (2022136). All the procedures were according to the principles of the Declaration of Helsinki. Informed consent was obtained from the participants and/or their legal guardians, and an opt-out approach was used because this was a retrospective observational study and there was no risk to the participants. Patients were provided with the opportunity to refuse participation in this study by posting their preferences on the institutional website (http://www.kmu.ac.jp/hirakata/hospital/2671t800000135zj.html). If the patients were below 20 years old, their parents or guardians had the right to refuse participation. All authors had access to the study data, and reviewed and approved the final manuscript.

## Results

### Clinical characteristics at the procedure of colonoscopies

Clinical characteristics in this study is showed in Table 1. Duration of disease was 6.2 years and approximately two thirds of patients had pancolitis in this cohort. Mean CRP was markedly high (5.0 mg/dL) and serum albumin levels was 3.0 mg/dL. Because the patients were suspected with CMV reactivation in this cohort, 65% of patients received anti-viral treatments while only 36% of patients received corticosteroids initially.

**Table 1:** Patient characteristics in patients who were suspected with cytomegalovirus (CMV) reactivation administered after admission.

Sex (male/female)	69/74
Age (years) (mean + /-SD)	52.1 ± 19.0
Disease duration (years) (mean + /-SD)	6.2 ± 12.6
pan colitis	87 (61%)
left-sided colitis	56 (39%)
PRO2 (mean + /-SD)	3.4 ± 1.7
CRP (mg/mL)	5.0 ± 5.8
Serum albumin level (mg/mL) (mean + /-SD)	3.0 ± 0.6
Mayo endoscopic score (mean + /-SD)	2.4 ± 0.8
Initial medical treatments after the hospitalization	
Corticosteroids	51(36%)
Advanced Therapy	23 (16%)
Anti-viral treatments	93 (65%)

PRO 2: Patients’ Report Outcome 2, CRP: C-reactive protein.

### Correlations between CMV colitis and endoscopic findings

To identify specific endoscopic findings of CMV colitis, the association between endoscopic characteristics and histological CMV positivity was analyzed. The sensitivities of punched-out and round ulcers for histological CMV positivity were 66.7% and 75.0%, respectively; however, the specificity and diagnostic accuracy were 58.3% and 46.6%, which might not be useful for predicting histological CMV positivity (Table 2). The specificity of wide mucosal defects for histological CMV positivity was 75.0%. However, few patients (2/12;16.7%) with wide mucosal defects showed histological CMV positivity. The diagnostic accuracies of endoscopic findings with punched-out, round, girdle, and longitudinal ulcers, and those with wide mucosal defects for histological CMV positivity were 59.1%, 49.2%, 84.0%, 41.7%, and 69.7%, respectively.

**Table 2:** Diagnostic accuracy of endoscopic findings for histological examination for CMV.

	Histological positive (+)	Histological positive (-)	Sensitivity	Specificity	PPV	NPV	Accuracy
Punched-out ulcer							
(+)	8	50	66.7%	58.3%	13.8%	94.6%	59.1%
(-)	4	70					
Round/oval ulcer							
(+)	9	64	75.0%	46.6%	12.3%	94.9%	49.2%
(-)	3	56					
longitudinal ulcer							
(+)	7	72	58.3%	40.0%	8.9%	90.6%	41.7%
(-)	5	48					
wide mucosal defect							
(+)	2	30	16.6%	75.0%	6.3%	90.0%	69.7%
(-)	10	90					

### Correlations between CMV viremia and endoscopic findings

Next, we assessed the the association between endoscopic characteristics and CMV antigenemia/ positivity of serum DNA test. The sensitivities of endoscopic findings with punched-out, round, and longitudinal ulcers for positivity of CMV antigenemia was intermediate (53.3%, 56.7%, and 56.7%), whereas specificities of these findings were low (Table 3a). In total, the diagnostic accuracies of endoscopic findings with punched-out, round, girdle, and longitudinal ulcers, and those with wide mucosal defects were 55.6%, 46.0%, 70.1%, 40.0%, and 60.4%, respectively. The sensitivities of endoscopic findings with punched-out, round, longitudinal ulcers, and wide mucosal defect for positivity of serum DNA test was 48.5%, 54.5%,63.6%, and 25.8%, whereas specificities of these findings were 43.8%, 18.8%, 25.0%, 56.3%, respectively (Table 3b). The specificity of girdle ulcers for positivity of serum DNA test was relatively high (84.8%) while sensitivity was 9.1%. In total, the diagnostic accuracies of endoscopic findings with punched-out, round, girdle, and longitudinal ulcers, and those with wide mucosal defects were 46.9%, 42.9%, 33.7%, 51.0%, and 35.7%, respectively.

**Table 3:** Diagnostic accuracy of endoscopic findings for a) CMV antigenemia and b) serum DNA qualitative test for CMV.

a)							
Endoscopic findings	C7HRP (+)	C7HRP (-)	Sensitivity,	Specificity	PPV	NPV	Accuracy
punched-out ulcer							
(+)	16	41	53.3	56.4	28.1%	79.1%	55.6%
(-)	14	53					
Round/oval ulcer							
(+)	17	54	56.7	42.5	23.9%	75.5%	46.0%
(-)	13	40					
Longitudinal ulcer							
(+)	17	61	56.7	35.1	21.8%	71.7%	40.0%
(-)	13	33					
Wide mucosal defect							
(+)	6	25	20.0	73.4	19.4%	74.2%	60.4%
(-)	24	69					
b)							
	Serum DNA qualitative test (+)	Serum DNA qualitative test (-)	Sensitivity	Specificity	PPV	NPV	accuracy
Punched-out ulcer							
(+)	32	18	48.5%	43.8%	64.0%	29.2%	46.9%
(-)	34	14					
Round/oval ulcer							
(+)	36	26	54.5%	18.8%	58.1%	16.7%	42.9%
(-)	30	6					
longitudinal ulcer							
(+)	42	24	63.6%	25.0%	63.6%	25.0%	51.0%
(-)	24	8					
wide mucosal defect							
(+)	17	14	25.8%	56.3%	54.8%	26.9%	35.7%
(-)	49	18					

### Comparison of endoscopic characteristics between patients who received ganciclovir and those who did not receive ganciclovir

Next, we compared endoscopic findings between patients who initially received GCV and patients without GCV. Among patients in this cohort, five patients who did not receive colonoscopy were excluded from this analysis regarding relationship between use of anti-viral treatments and endoscopic findings. The proportions of cases with punched-out ulceration (57% vs. 13%, p < 0.001), round/oval ulceration (65% vs. 32%, p < 0.001), longitudinal ulceration (70% vs. 34%, p < 0.001), and ulceration with wide mucosal defects (33% vs. 9%, p < 0.001) were significantly higher in cases where ganciclovir was used than in cases without ganciclovir (Table 4). The mean MES and UCEIS scores were also significantly higher in patients with ganciclovir (p < 0.001). Of note, only erosion/ulceration score of UCEIS (p < 0.001), not vascular score (p = 0.300), bleeding score (p = 0.629) was significantly higher in cases with ganciclovir (2.3 ± 0.9) than cases without ganciclovir (1.6 ± 0.9) (Table 4).

**Table 4:** Endoscopic severity and characteristics in patients who were treated with GCV (GCV group) or without GCV (no GCV group). Patients who did not receive colonoscopy were excluded (2 patients of GCV group and 3 patients of no GCV group).

	Initial GCV (n = 91)		no GCV (n = 47)		p value
punched-out ulcers	52	57%	6	13%	p < 0.001
round ulcers/ oval ulcers	59	65%	15	32%	p < 0.001
annular ulcers/ girdle ulcers	10	11%	3	6%	P = 0.292
longitudinal ulcers	64	70%	16	34%	p < 0.001
wide mucosal defect	28	31%	4	9%	p < 0.001
MES	2.6 ± 0.6		2.0 ± 0.8		p < 0.001
UCEIS	5.9 ± 1.6		5.0 ± 1.8		P = 0.004
UCEIS/ Vascular pattern	1.9 ± 0.3		1.9 ± 0.3		p = 0.328
UCEIS/ Bleeding	1.7 ± 0.9		1.7 ± 0.9		p = 0.789
UCEIS/ Erosions and Ulcers	2.3 ± 0.9		1.6 ± 0.9		p < 0.001
PRO2	3.7 ± 1.2		4.0 ± 1.5		p = 0.300
CRP	5.7 ± 6.0		5.2 ± 5.8		p = 0.629
Serum albumin	2.9 ± 0.6		3.4 ± 0.5		p < 0.001

MES: Mayo Endoscopic subscore, UCEIS: Ulcerative colitis endoscopic index of severity, PRO 2: Patients Report outcome 2, CRP: C-reactive protein,

## Discussion

CMV colitis occurs in immunocompromised patients, and colonoscopic findings related to CMV infection have been investigated in patients with acquired immunodeficiency syndrome and transplant recipients [21–23]. Although deep ulceration is a typical finding for CMV infection, identifying the characteristics of CMV colitis associated with UC is considered challenging because ulceration is found in most cases of severe UC. Considering this, research has been conducted on the characteristic endoscopic findings of CMV colitis. Nishimoto et al. reported CMV colitis in patients with UC and described that well-demarcated ulcers may be a sign of CMV infection in such patients [24]. Previous studies have suggested that irregular ulceration, wide mucosal defect, punched-out ulceration, and longitudinal ulceration were predictive factors for CMV colitis [15–17]. CMV is recognized as an important pathogen in immunocompromised patients. In patients with UC, CMV reactivation is a factor that exacerbates the condition, and clinical outcomes are poor in these patients [25–27]. Previous research has also indicated that evidence of CMV colitis results in higher colectomy rates [28,29]. Consequently, the therapeutic management of patients with UC with CMV reactivation is of utmost importance. However, the role of CMV in triggering clinical recurrence, acute severe colitis, and treatment resistance in patients with CMV reactivation remains unclear [30]. While a previous study indicated that immunosuppressive therapy plays a significant role in the risk of CMV reactivation, it is debatable whether CMV worsens UC inflammation or is merely present in the inflamed mucosa. If CMV reactivation is responsible for worsening UC inflammation, antiviral treatment should be used. On the other hands, if it is merely present then medical treatment for UC should be initially selected.

Accurate diagnosis of CMV infection is critical for patients with acute severe UC, and methods such as immunohistochemistry and serum/mucosal PCR for CMV reactivation have been proven useful. The sensitivity and specificity of immunohistochemistry for detecting CMV reactivation are high [31], but colonic mucosal assessment of CMV takes a relatively longer time. Moreover, serum or mucosal PCR is not available at general institutions or in the clinical real world. These challenges had prompted research to predict CMV infection based on endoscopic findings, such as punched-out ulceration as described above. Based on these findings, some Japanese institution adopted the practice of administering antiviral treatment before initiating corticosteroid treatment for active UC until recently. In fact, our cohort showed that ganciclovir was frequently used as an initial treatment in patients with severe disease and patients with punched-out ulcers or longitudinal ulcerations because a few advanced therapies could be used until a recent decade. Patients who had severe endoscopic findings such as deep ulceration, longitudinal ulceration and wide-mucosal defect more frequently selected anti-viral treatment as an initial treatment after hospitalization in our study (Table 4). Endoscopic diagnostic accuracy for CMV reactivation, however, had not been demonstrated well. Therefore, we analyzed endoscopic diagnostic accuracy of typical endoscopic findings (punched-out ulcer, round ulcer) in patients who were suspected with CMV reactivation in the clinical setting. Our study indicated there were few cases of CMV antigenemia positivity or histological CMV positivity in patients with typical endoscopic findings such as deep ulceration with wide mucosal defect. These results suggest that endoscopic findings cannot predict CMV antigenemia or CMV colitis.

The previous Japanese guidelines (until 2020) for the treatment of UC from the Health and Labor Sciences Research Grants for Research on Intractable Diseases from the Ministry of Health, Labour and Welfare of Japan indicated that a typical endoscopic finding in cases of CMV infection was the formation of circular and punched-out ulcers. Additionally, the combination of antiviral drugs may be effective in cases of CMV enteritis. According to these descriptions, antiviral drugs may be administered in the absence of evidence of CMV reactivation in serum or intestinal tissue in some institutions, including our institution. However, based on the results of the present study, the diagnostic ability of the characteristic endoscopic findings of CMV enteritis was not satisfactory. Therefore, we believe that the use of antiviral drugs based solely on endoscopic findings should be avoided. More recently, the 2024 Japanese treatment guidelines stated that punched-out ulcers may be observed even during the active stage of UC, and that CMV infection cannot be diagnosed based on endoscopic findings alone. Our results are consistent with this statement.

Our study had some limitations. First, this was a single-center retrospective study and number of patients was not large. Second, the comparison between the patients with and without GCV involved a selection bias because the endoscopic findings influenced the treatment selection itself. Third, mucosal PCR for CMV was not performed in our study. Mucosal PCR may be useful in diagnosing CMV infection in patients with UC [32], but it is not generally performed in clinical settings. According to the recent guideline, immunohistochemistry or PCR on gastrointestinal tissue is recommended for the diagnosis of acute CMV colitis, since serum antigenemia and PCR tests do not necessarily correlate with colonic mucosal infection [33]. A recent study by Ye et al. also demonstrated that the proportion of patients with punched-out, longitudinal, or wide mucosal ulcerations was significantly higher among those with positive mucosal CMV PCR results compared to those with negative results [34]. This finding suggests that localized mucosal CMV infection may be reflected by characteristic endoscopic features. However, even in that study, more than half of the patients with deep or longitudinal ulcers were negative for CMV by tissue PCR. This observation implies that sampling limitations and the focal nature of CMV infection may lead to false-negative results. Therefore, the negative results in our study may reflect these methodological and practical constraints rather than the true absence of mucosal CMV infection.

In conclusion, the diagnostic accuracy of endoscopic findings for cytomegalovirus reactivation in hospitalized patients with ulcerative colitis is not satisfactory. Therefore, antiviral treatment should not be administered solely based on endoscopic findings without clear evidence of CMV reactivation.

## Supporting information

S1 Data Data sets. (XLSX)
